# Developing a model osteoarthritis consultation: a Delphi consensus exercise

**DOI:** 10.1186/1471-2474-14-25

**Published:** 2013-01-16

**Authors:** Mark Porcheret, Janet Grime, Chris Main, Krysia Dziedzic

**Affiliations:** 1 Arthritis Research UK Primary Care Centre, Keele University, Keele, Staffordshire, ST5 5BG, United Kingdom

**Keywords:** Primary care, General practice, Osteoarthritis, Patient-centred care, Physician-patient relationship, Health services research

## Abstract

**Background:**

Osteoarthritis (OA) is a common condition managed in general practice, but often not in line with published guidance. The ideal consultation for a patient presenting with possible OA is not known. The aim of the study was to develop the content of a model OA consultation for the assessment and treatment of older adults presenting in general practice with peripheral joint problems.

**Methods:**

A postal Delphi consensus exercise was undertaken with two expert groups: i) general practitioners (GPs) with expertise in OA management and ii) patients with experience of living with OA. An advisory group generated 61 possible consultation tasks for consideration in the consensus exercise. Expert groups were asked to consider which tasks should be included in the model OA consultation. The exercise was completed by 15 GPs and 14 patients. The level of agreement for inclusion in the model was set at 90%.

**Results:**

The model OA consultation included 25 tasks to be undertaken during the initial consultation between a GP and a patient presenting with peripheral joint pain. The 25 tasks provide detailed advice on how the following elements of the consultation should be addressed: i) assessment of chronic joint pain, ii) patient’s ideas and concerns, iii) exclusion of red flags, iv) examination, v) provision of the diagnosis and written information, vi) promotion of exercise and weight loss, vii) initial pain management and viii) arranging a follow-up appointment. Both groups prioritised a bio-medical approach to the consultation, rather than a bio-psycho-social one, suggesting a discordance between current thinking and research evidence.

**Conclusions:**

This study has enabled the priorities of GPs and patients to be identified for a model OA consultation. The results of this consensus study will inform the development of best practice for the management of OA in primary care and the implementation of evidence-based guidelines for OA in primary care.

## Background

Osteoarthritis (OA) is a highly prevalent condition which presents and is managed in primary care [1]. Evidence-based guidelines on its management have been published by professional bodies and the UK National Institute for Health and Clinical Excellence (NICE) [2-7]. The NICE OA Guideline recommends: i) a holistic approach to the management of OA ii) three core treatments (access to information, exercise and physical activity and interventions to achieve weight loss) be offered to all people with OA and iii) a range of other evidence-based interventions for those with persisting pain and/or disability [6]. Evidence suggests that management of patients presenting with OA in the UK is not in line with published guidance: older patients consulting with peripheral joint pain report that the problem may be dismissed [8,9] and NICE core treatments are not routinely offered early on in the course of the condition [10-12]. This paper forms part of a wider study investigating how to improve implementation of NICE OA guidance in UK primary care.

What then are the potential components of an ideal consultation for OA? To investigate this we have undertaken a consensus exercise to determine the views of patients and clinicians about the possible content of an “ideal” consultation between a GP and a patient presenting with joint pain. Although there are evidence-based frameworks for medical consultations, notably the Calgary-Cambridge Framework [13], and there is the background science about primary care OA management summarised in the NICE guidelines, there is no empirical evidence to guide the identification of the specific content of a model OA consultation. In such circumstances, consensus studies with experts have been advocated as the “next best” option [14,15]. The relevant “experts” are the two participants in the consultation, namely, in the UK, the general practitioner (GP) and the patient presenting with OA [16].

The aim of the consensus exercise was to elicit the views of a GP group and a patient group (patients who have OA) on the content of a model OA consultation and determine consensus about which specific tasks might be included in such a consultation.

## Methods

A Delphi consensus exercise [17] was undertaken in four stages: i) an ideas generation round, ii) development of a common consensus questionnaire for GP and patient groups, iii) consensus rounds undertaken separately by the groups and iv) establishing the criterion for consensus in terms of the level of agreement needed for a statement to be included in the model OA consultation. Ethical permission for the study was given by South Manchester Research Ethics Committee (reference number: 09/H1003/2).

### Stage 1 - ideas generation round

#### Initial development

The NICE Guideline for the management of OA, was used as the basis for a set of principles which were specified in advance of the consensus exercise: i) a primary care OA consultation needed to cover the focused assessment of an older adult presenting with a peripheral joint problem and the consequent treatment of those considered to have OA. ii) the consultation would be patient-centred and support self-management, iii) the diagnosis of OA would be made clinically, iv) if a diagnosis of OA was made the GP would offer the patient written information about OA (an OA Guidebook which had been developed for the study investigating the implementation of the NICE OA Guideline referred to above), and v) the treatment algorithm advocated in the NICE OA guideline would be followed. Further, vi) follow-up appointments with a specially trained healthcare professional would be routinely available to further support self-management of osteoarthritis, and would be offered during the consultation (a service being provided in the implementation study) and vii) the Calgary-Cambridge model was chosen as the framework for the consultation. The Calgary-Cambridge model consists of 71 consultation skills which clinicians should be able to utilise when communicating with patients, which are organised in a framework describing the flow of a typical consultation: initiating the session, gathering information, physical examination, explanation and planning, and closing the consultation. It is very widely used in Medical School communication skills teaching and underpins the UK’s Royal College of General Practitioners curriculum for the consultation [18].

Prior to the consensus exercise the research team drew up an initial list of 34 statements about a model OA consultation based on the processes listed in the Calgary-Cambridge framework.

#### The advisory group

Inclusion criteria for the advisory group were: i) professionals who were expert in the management of OA or ii) lay people who were “expert” in what it is like to have the condition. Membership was invited from: i) former members of the NICE OA Guideline Development Group, ii) members of the Arthritis Research UK Primary Care Centre and iii) members of the Arthritis Care Helpline team. A group of 27 professionals (ten GPs, five physiotherapists, four rheumatologists, three nurses, three occupational therapists and two social scientists) and seven lay people was identified.

The initial list of 34 statements developed by the research team was sent to members of the advisory group, who were asked to comment on each statement and to suggest additional statements. The comments and suggested additions were collated, and reviewed for consistency and overlap, and a final list of 61 statements was developed for consideration in the consensus rounds.

### Stage 2 - consensus questionnaire

The questionnaire consisted of a case scenario, task instructions, the final list of statements and a consent form. The patient presented in the scenario had a problem with their knee:

● A 57 year old attends the GP for the first time with a knee problem. The problem has worsened over the past few months and the patient has come to ask for help coping with it.

The GPs and patients undertaking the consensus exercise were given a framework for the consultation which was based on the set of principles listed in stage 1. The instructions stated that: i) the treatment algorithm in the 2008 NICE OA Guideline should be followed, and a figure of the NICE target algorithm was included; ii) the consultation was to support the patient’s self-management of OA; iii) the diagnosis would be made clinically; iv) if a diagnosis of OA was made the GP should offer the OA Guidebook and a follow-up appointment with the specially trained healthcare professional. To simplify the consensus task, the scenario focused on one joint, the knee, and the tasks to be considered were those for the assessment and treatment of a problem in the knee rather than any peripheral joint. Participants were asked to consider the statements regarding explanation and planning on the basis that a diagnosis of OA had been made and that the OA Guidebook would be given to the patient.

### Stage 3 - consensus rounds

#### Sample size calculation and recruitment of expert groups

A consensus methods review [14] suggested that consensus groups should have between 6 and 12 members. If fewer than this, reliability declines, whereas little further is gained by having more than 12 contribute to the final consensus round. Assuming a 70% response to each round (60% for GPs), and two consensus rounds, sample sizes needed for the two consensus groups were calculated as: patient group (n = 25), GP group (n = 35).

The inclusion criteria were: for the GP group, expertise in managing OA; for the lay group, having, or caring for someone with, OA. Potential members of the GP group were recruited at the 2008 Primary Care Rheumatology Society Annual Conference. Recruitment of the patient group was undertaken by inviting members of the Research User Group at the Arthritis Research UK Primary Care Centre and previous participants in a Centre study, to join the group. All persons indicating a willingness to participate in the study were sent the first and second consensus questionnaires by post, and non-responders to either round were sent a reminder at two weeks. No payments were made for participation in the study.

#### Composition and characteristics of expert groups

32 GPs and 23 patients expressed an interest in participating in the study and were mailed the round 1 questionnaire. 16 GPs and 14 patients returned a round 1 questionnaire and of these all bar one GP completed and returned a round 2 questionnaire, a round 2 response of 47% and 61% respectively.

The GPs all declared a special interest in musculoskeletal disorders and were predominantly established GPs. The members of the patient group had a mean age of 72 years (interquartile range 67–76 years) and all had, or were caring for a person with, OA. Group characteristics are shown in Table 1.

**Table 1:** Characteristics of GP and patient groups

**Characteristic**	**Number (%) group members**
**GP Group (n = 15)**	
Female	6 (40)
Qualified as a GP for 5 yrs or longer	12 (80)
Undertakes dedicated musculoskeletal sessions	11 (73)
Practice type – urban/rural/mixed	10 (67)/1 (7)/4 (26)
Practice list size greater than 7 000	10 (67)
Undergraduate or postgraduate training practice	14 (93)
**Patient Group (n = 14)**	
Female	6 (43)
Reported “having osteoarthritis”	13 (93)
Reported ever consulting for osteoarthritis	11 (79)
Reported caring for someone with osteoarthritis	3 (21)

#### Undertaking consensus rounds

In the first consensus round participants were asked to decide which statements should be included if “time was no object” (for example, if there was an extended period of time for the consultation or if it could be conducted over several appointments). Participants were asked to rate each statement using a five-point Likert scale (definitely include/probably include/undecided/probably not include/definitely not include) as anchors and a “don’t know” option.

In the second round participants were asked to consider which statements should be included if the consultation was only 10 minutes long (the normal maximum for GP consultations in the UK). For each statement participants were fed back their individual response from the first round and the total number of responses by their group for each item, and were asked to re-rate the statements.

The decision to define the length of the consultation differently in rounds 1 and 2 was made for pragmatic reasons: it was felt too onerous for participants in round 1 to decide which statements, from an extensive list, they would include in a time-limited consultation, and so a two-stage approach was adopted.

#### Analysis of round 2 responses

The responses from the patient and GP groups in the second consensus round were analysed separately, but using the same methodology. The proportion of participants who responded to each Likert item was calculated for each statement. Participants who had responded “don’t know”, or for whom there was missing data, were excluded from the denominator for the relevant statement. A response of either “definitely include” or “probably include” was defined as a response to include the statement in the model OA consultation.

### Stage 4 - defining consensus

The level of agreement used to define consensus is often arbitrary [14]. Some studies have “set the bar” for agreement at the level of a simple majority, while others have set the bar higher [14]. We wanted to identify a set number of consultation tasks which could realistically be undertaken in a 10-minute consultation, and not to pre-define an arbitrary level of agreement for a task to be included in the model OA consultation. For this reason an analysis of the number of statements at different levels of agreement for inclusion was undertaken to consider where to “set the bar”.

#### Number of statements by level of agreement

The GP group demonstrated a high level of agreement for inclusion for many of the statements (Table 2). The patient group had a high level of agreement for fewer statements (Table 2). The cumulative number of statements which would be included at differing levels of agreement was determined for both groups (Table 2).

**Table 2:** Number of statements by level of agreement and cumulatively included for consensus groups

**Level of agreement for inclusion (%)**	**GP Group**			**Patient Group**		
	**No. of statements**	**Cumulative level of agreement (%)**	**No. of statements cumulatively included**	**No. of statements**	**Cumulative level of agreement (%)**	**No. of statements cumulatively included**
100	11	100	11	2	100	2
90 – 99	14	> = 90	25	4	> = 90	6
80 – 89	4	> = 80	29	5	> = 80	11
70 – 79	5	> = 70	34	10	> = 70	21
60 – 69	8	> = 60	42	16	> = 60	37
50 – 59	3	> = 50	45	9	> = 50	46
<50	16			15		

#### “Setting the bar”

The bar was set at the same level for both groups and a statement was included if either (or both) group included it at or above the level of the bar. If the bar was set at 100% then 11 statements would be included. If the bar was lowered to 90% then a further 14 statements would be included in the model OA consultation resulting in 25 tasks in total. Lowering the bar to 80% would add an additional five statements resulting in 30 tasks being included in the model OA consultation.

From this analysis, it was felt that if the bar was set at 100% fewer tasks (11 tasks) than could be comfortably undertaken in a 10-minute consultation would be included, but setting it at 90% a realistically do-able number of tasks (25 tasks) would be included. Lowering the bar further would increase the number of tasks to be included and would result in more tasks being included than could realistically be undertaken in 10 minutes. For this reason it was decided to set the bar at 90% - a high level of agreement at which the number of tasks included could be realistically undertaken in a 10-minute consultation.

## Results

The 25 tasks with a level of agreement of 90% or more in either or both groups included in the model OA consultation are shown in Table 3. The tasks are those which were prioritised for inclusion in a 10-minute consultation between a GP and a patent presenting with chronic joint pain and enable detailed advice to be given as to how GPs could approach such a consultation. The first 12 tasks in the model detail the preferred approach by the groups to taking the history and examining the patient. The rest of the tasks give advice on the approach to giving and explaining the diagnosis, providing support for self-management and addressing the patient’s need for analgesia. The two tasks given the highest priority were: i) enquiry about the need patient’s need for painkillers and ii) recommending paracetamol and/or topical NSAIDs to address this need. There was 100% agreement for inclusion of these two tasks by both groups.

**Table 3:** Statements for inclusion in the model OA consultation

**Statement**^1^	**No. (%) GP Group would include (n = 15)**	**No. (%) Patient Group would include (n = 14)**
**The GP:**^2^		
Encourages the patient to give a full account of the problem(s), including the reason for coming today	15 (100)	11 (79)
Finds out how long the patient has had the knee problem for and whether the problem comes and goes	14 (93)	12 (86)
Asks specific questions about the amount and type of any pain	14 (100)	11 (79)
Asks about other knee symptoms such as stiffness, locking and giving way	13 (93)	12 (86)
Asks about problems with mobility, such as walking, going up and down stairs, and getting in and out of a chair	13 (93)	9 (64)
Asks if, and how, the knee problem affects activities such as work, hobbies, sports and general leisure activities	14 (100)	7 (50)
Asks about previous problems with the knee, knee operations, knee injections	13 (93)	11 (79)
Asks about problems with other joints, especially the other knee and the hips	14 (93)	8 (62)
Asks about the patient’s ideas, concerns, fears and feelings about the problem	14 (93)	7 (54)
**Asks if the patient has tried anything to help the problem, and if yes, what/how used/how effective**	**15 (100)**	**12 (92)**
Checks if there is anything in the patient’s story to suggest a fracture, cancer, inflammatory or septic arthritis	14 (93)	7 (54)
Examines the knee joint and surrounding tissues	15 (100)	11 (85)
**Informs the patient that the most likely reason for the problem is osteoarthritis and explains the reason(s) for coming to this diagnosis**	**15 (100)**	**12 (92)**
**Gives a brief explanation of osteoarthritis**	**14 (93)**	**12 (92)**
Asks if the patient has any unanswered questions	15 (100)	8 (57)
Hands the guidebook to the patient with the advice to read it	14 (93)	8 (62)
Encourages the patient to consider the use of “NICE core treatments”, increased physical activity/muscle strengthening exercises/dietary changes to lose weight, if needed	14 (93)	10 (77)
Emphasises, when relevant, the benefit of losing weight: that if weight is lost then the pain reduces	14 (93)	10 (77)
Emphasises, when relevant, the benefit of exercise in helping to lose weight in addition to the benefits for osteoarthritis	14 (93)	8 (62)
**Enquires about the patient’s need for painkillers**	**15 (100)**	**13 (100)**
**Recommends the use of paracetamol and/or topical NSAIDs (creams or ointments) before the use of other painkillers**	**15 (100)**	**13 (100)**
Summarises the management plan and re-checks that it is acceptable to the patient	14 (93)	9 (64)
**Advises the patient to make a follow up appointment with the specially trained healthcare professional**	**15 (100)**	**13 (93)**
Uses free-text to record the consultation in the paper/electronic records	14 (93)	8 (67)
In addition to statement above records coded data on the; i) diagnosis and ii) main elements of the consultation, such as the level of pain, the BMI and advice to exercise	15 (100)	10 (77)

**1 Statement in bold if 90**% **or more agreement in BOTH groups.**

2 “The GP” is the stem for all the statements.

With a level of agreement for inclusion set at 90% or more, 36 statements were excluded from the model OA consultation (Table 4).

**Table 4:** Statements excluded from the model OA consultation

**Statement**	**No. (%) GP group would include (n = 15)**	**No. (%) Patient group would include (n = 14)**
The GP:*		
Assesses the degree of pain using a formal measure, such as rating the pain on a scale from 0 to 10	1 (7)	8 (57)
Assesses the extent of mobility problems using a formal measure, such as a rating scale from 0 to 10.	1 (7)	7 (50)
Asks about a family history of joint problems	6 (43)	4 (29)
Asks about jobs which may have affected/caused the knee problem, such as those involving a lot of kneeling (for example, carpet fitter, cleaner, joiner, electrician)	9 (64)	5 (36)
Asks about the patient’s expectations of the consultation	10 (67)	4 (31)
Asks which problem, concerning the knee, the patient wants help with most, for example pain, stiffness or climbing the stairs	9 (60)	5 (38)
Asks about who the patient has seen, or asked for help from, about the problem	10 (71)	6 (46)
Assesses the patient’s mood for symptoms of anxiety and depression	8 (53)	1 (8)
Screens the patient for depression using a formal depression screening tool	0 (0)	0 (0)
Asks about other conditions, such as diabetes, heart or kidney disease, which might affect the management of the knee problem	10 (67)	9 (64)
Asks about circumstances, such as unemployment and financial hardship, which might affect the management of the knee problem	5 (33)	0 (0)
Assesses the knee joint by general observation of the patient’s walking pattern, mobility and footwear	13 (87)	9 (69)
Performs a specific test, such as a timed walk test, to assess function	0 (0)	3 (21)
Examines the other knee, hips and hands for signs of osteoarthritis	11 (73)	10 (71)
If not recently done, measures weight and height to calculate the body mass index	6 (40)	6 (46)
Undertakes a full examination of the locomotor system (of the joints and muscles)	0 (0)	4 (33)
Enquires about the patient’s views and understanding of osteoarthritis	13 (87)	9 (75)
In addition to giving a brief explanation explains the likely cause of osteoarthritis	4 (27)	9 (69)
In addition to giving a brief explanation explains the likely outcome for people with osteoarthritis	9 (60)	8 (62)
Explores the patient’s understanding of the information given, and their reaction/beliefs/feelings about it	8 (53)	8 (62)
Tells the patient that they are central to the management of their own condition: that self-management of osteoarthritis is necessary and important	13 (87)	11 (85)
Explains that the central role of the primary healthcare team in the management of osteoarthritis is to support and guide self-management	7 (47)	9 (69)
Explains the purpose of managing osteoarthritis to: improve understanding, reduce pain, improve mobility and reduce the risk of it getting worse	9 (60)	12 (86)
Explains the approach to the treatment of osteoarthritis recommended by NICE	3 (20)	8 (62)
In addition to handing out the guidebook highlights sections in the guidebook relevant to the patient’s problem	6 (40)	6 (46)
Asks if the patient has any views/preferences for what treatment they might want to consider next, and, if they do, what they are	12 (80)	6 (43)
Takes an “exercise history”: the patient’s attitude to taking exercise/physical activity/exercises and their experience of these	9 (60)	6 (43)
Takes a “weight history”: the patient’s attitude to losing weight and their prior experience of doing this	7 (47)	9 (69)
Indicates, if the patient is overweight, where they are on a body mass index chart	7 (47)	9 (69)
Explains that exercise may cause muscle soreness initially and that the benefits of exercise may not be immediate	9 (60)	5 (38)
Explains the risks and benefits of painkillers	11 (73)	6 (50)
Discusses with the patient whether any other extra treatment needs to be considered	7 (47)	8 (67)
Discusses appropriate referrals, for example to; physiotherapy, occupational therapy, podiatry, social services, community pharmacy, district nursing service or work support services	8 (53)	10 (71)
Discusses the option of joint replacement surgery in patients with established severe pain, or severe functional limitation, in addition to core treatments and painkillers	7 (47)	7 (54)
Formulates with the patient a self-management plan	11 (73)	10 (77)
Explains when the patient should re-consult the GP	11 (73)	8 (57)

* “The GP” is the stem for all the statements.

## Discussion

### Summary of main findings

Setting the bar for consensus at 90% resulted in the identification of 25 consultation tasks to be undertaken during the initial consultation between a GP and a patient presenting with peripheral joint pain. The 25 tasks provide detailed advice on how the following elements of the consultation should be addressed: assessment of chronic joint pain, patient’s ideas and concerns, exclusion of red flags, examination, provision of the diagnosis and written information, promotion of exercise and weight loss, initial pain management, and arranging a follow-up appointment. There was high level of agreement in the GP group to include many of the tasks proposed for the model consultation; the patient group had high levels of agreement for fewer tasks.

### Comparison with existing literature

The items included in the consensus study for the model OA consultation cover both the assessment of the problem and its treatment if a diagnosis of OA has been made and is to the authors' knowledge the first study using consensus methodology to characterise such a consultation. Two trials [19,20] have previously evaluated the effect of a standardised approach to consulting for OA. One of these [19] included both assessment and treatment, but in both studies the content of the consultation was developed by a group of experts through discussion and reference to published guidelines, and the methodologies for these have not been published. Standard textbooks on clinical methods [21,22] are focused primarily on the examination rather than history taking and do not cover in detail the assessment of peripheral joints in older people. A textbook on the 10-minute clinical assessment [23] includes, in the section on the assessment of knee pain, many of the tasks with a high level of agreement for inclusion in our model OA consultation such as eliciting ideas and concerns, taking a “pain history” and understanding the effect of the problem on mobility and work.

The two tasks given the highest priority, those which all the participants from both groups included, concerned the pharmacological management of pain. However, they did not prioritise psycho-social tasks such as assessing mood and asking about social circumstances, suggesting that both groups favoured a bio-medical approach to the initial consultation rather than a biopsychosocial one. This suggests a discordance between “current thinking” of practising GPs, and patients, and “current best thinking” from research evidence, which suggests that an integrated biopsychosocial approach should be adopted for OA [6]. Possible reasons for this discordance might be; the dominance of the practicalities of achieving something in the 10 minutes of a consultation, the GPs’ lack of awareness of this research, the influence of the prevalent bio-medical approach to osteoarthritis [24,25], that the relevance of psychosocial management to clinical management of OA has yet to be established or GPs’ perceptions of clinical priorities in a first consultation for such a problem. Concerning the last point, similar patient views supporting a biomedical approach for initial consultations for a problem have been identified previously in other clinical areas; for example Calnan et al [26]. found that patients’ explanations for upper limb disorders were initially biomechanical, with psychosocial explanations only being invoked when these were no longer appropriate. Neither of the two groups in our study prioritised tasks eliciting patient expectations, which is counter to a patient-centred approach propounded in the biopsychosocial approach, or in current notions of the “patient-as-person”, sharing power and responsibility and therapeutic alliance [27].

### Strengths and limitations

The inclusion of patients in the consensus exercise represents a particular strength of this study. The levels of agreement for the statements were lower and more varied in the patient group than the GP group and, by “setting the bar” at the same level for both groups, the GP group contributed more tasks to the model than the patient group. However, the majority of the patient group was in favour of including all the 25 consultation tasks in the model and lowering the bar in the patient group to 80% would only have included two additional tasks. The response in the GP group was low, but this was in line with responses in other studies with GPs as participants [28] and still resulted in 15 GPs completing the consensus exercise, a number which has been shown to be sufficient for such exercises [14]. The participating GPs may not have the same views as GPs as a whole, as they all declared a special interest in musculoskeletal disorders, but it does seem reasonable to use the views of “specialist” GPs when evidence suggests that GPs in general have not fully engaged with the management of OA.

The tasks which the consensus groups prioritised produced a model that had a bio-medical focus and was not fully patient-centred – “eliciting patient expectations” for example was not included - and obtaining this result could be seen as a weakness of a methodology to develop a patient-centred consultation. However, the patient group could have, but did not, prioritise “patient expectations”, suggesting that for this group such an aspect of the consultation was not an essential feature of patient centredness, and our aim was to elicit consensus around current views of patients and professionals on consulting for OA as an important starting point when planning how to implement change.

### Implications for future research and clinical practice

The consensus exercise was undertaken in the context of the development of an approach to the management of OA to be used in an intervention study investigating how to implement best primary care for OA. The consensus we have obtained will form part of the framework, together with other clinical, scientific, guideline and policy evidence, in shaping the final content of a model for the initial consultation between a GP and a patient presenting with peripheral joint pain for use in the implementation study. These insights from the consensus exercise, into current GP and patient opinion on priorities for such a consultation will be used to inform the development of the training programme in the study.

More generally, the results of this consensus study can inform primary care training for OA management. Although the context of day-to-day practice is different from that used in the consensus exercise, for example the provision of a specially trained healthcare professional to support the self-management of OA is not generally provided in clinical practice currently, many of the tasks which were identified for inclusion in the model OA consultation do not rely on such a service being available and would be relevant to current clinical practice.

## Conclusion

This study has identified current consensus of a group of GPs and patients on the content of a model OA consultation for primary care. Overall 25 tasks, covering assessment and initial management of OA, were identified for inclusion in the model. The model OA consultation will need to be shaped for use in clinical practice and for investigating how to implement the NICE OA Guidelines in practice.

## Competing interests

The authors have no competing interests to declare.

## Authors’ contributions

All authors have made substantial contributions to the development of the study, analysing the data, drafting and revising the manuscript and have given final approval for this version to be published. In addition, MP carried out the data collection for the study.

## Authors’ information

M Porcheret, Senior Lecturer in General Practice, FRCGP MPhil; J Grime, Social Science Researcher (retired), MMedSci; C Main. Professor of Clinical Psychology (Pain Management), PhD: K Dziedzic, Arthritis Research UK Professor of Musculoskeletal Therapies, PhD.

## Pre-publication history

The pre-publication history for this paper can be accessed here:

http://www.biomedcentral.com/1471-2474/14/25/prepub
